# Challenges in Abdominal Organ Transplantation During the COVID-19 Pandemic

**DOI:** 10.3389/fmed.2020.00287

**Published:** 2020-06-04

**Authors:** Stepan M. Esagian, Ioannis A. Ziogas, Dimitrios Giannis, Muhammad H. Hayat, Nahel Elias, Georgios Tsoulfas

**Affiliations:** ^1^Surgery Working Group, Society of Junior Doctors, Athens, Greece; ^2^Department of Surgery, Division of Hepatobiliary Surgery and Liver Transplantation, Vanderbilt University Medical Center, Nashville, TN, United States; ^3^Institute of Health Innovations and Outcomes Research, The Feinstein Institute for Medical Research, Manhasset, NY, United States; ^4^Department of Medicine, Division of Gastroenterology, Hepatology and Nutrition, Vanderbilt University Medical Center, Nashville, TN, United States; ^5^Department of Surgery, Transplantation Unit, Massachusetts General Hospital and Harvard Medical School, Boston, MA, United States; ^6^First Department of Surgery, Aristotle University of Thessaloniki, Thessaloniki, Greece

**Keywords:** coronavirus, liver transplantation, kidney transplantation, immunosuppression, super-spreading events

## Abstract

As the coronavirus disease 2019 (COVID-19) outbreak has rapidly evolved into a global pandemic, abdominal organ transplantation programs are currently facing multiple challenges. Transplant candidates and recipients are considered high-risk populations for severe disease and death due to COVID-19 as a result of their numerous underlying comorbidities, advanced age and impaired immune function. Emerging reports of atypical and delayed clinical presentations in these patients generate further concerns for widespread disease transmission to medical personnel and the community. The striking similarities between COVID-19 and other outbreaks that took place over the past two decades, like Severe Acute Respiratory Syndrome and Middle East Respiratory Syndrome, highlight the severity of the situation and dictate that extra measures should be taken by the transplant programs to avoid adverse outcomes. Transplant organizations are currently calling for strict screening and isolation protocols to be established in all transplant programs, for both organ donors and recipients. As the situation escalates, more radical measures might be necessary, including a temporary hold on non-urgent transplantations, resulting in serious ethical dilemmas between the survival of these patients and the safety of the community. Further data about these special populations could result in more individualized guidelines for abdominal organ transplantation in the era of COVID-19.

## Introduction

In late December 2019, a series of pneumonia of unknown origin emerged in the city of Wuhan, China. The pathogen was identified to be a novel enveloped RNA betacoronavirus named SARS-CoV-2 (Severe Acute Respiratory Syndrome coronavirus 2) (1). The viral disease, named COVID-19 (coronavirus disease 2019), was declared as a pandemic by the World Health Organization on March 11th, 2020 (2). By May 21st, 2020, there were over 4,800,000 confirmed cases, more than 320,000 deaths attributed to the disease, and 216 countries and territories have been affected worldwide (3). The novel coronavirus is associated with a high risk of acute respiratory disease and Intensive Care Unit (ICU) admission (4, 5). It is hard to project the future dynamics of this pandemic and its long-term impact on worldwide healthcare. In this mini review, we aimed to examine the potential effects of the COVID-19 pandemic on abdominal organ transplantation.

## Clinical Characteristics and Outcomes—Are Abdominal Organ Transplant Recipients and Candidates at Increased Risk?

According to a report by the Chinese Center of Disease Control and Prevention (CCDC), the manifestations of SARS-CoV-2 laboratory-confirmed infection varied from asymptomatic/mild disease (81%) to severe disease (14%), and critical disease (5%) (6). The clinical presentation most commonly consists of fever (>85%), cough (>65%), myalgia, or fatigue (>40%) (4, 5, 7). A minority of patients (<15%) develop headache, confusion, and chills, while gastrointestinal symptoms (nausea, vomiting, and diarrhea) are less common (4, 7). Common imaging findings include bilateral patchy shadowing on chest radiography and ground glass appearance on computed tomography, while lymphocytopenia (>80%) was the most characteristic laboratory finding (7). According to the report of the first case series from China, a significant proportion of patients (23.7%) suffered from comorbidities, which are commonly seen in abdominal transplant candidates, including hypertension (15.0%), diabetes mellitus (7.2%), hepatitis B infection (2.1%), cancer (0.9%), chronic kidney disease (0.7%) and immunodeficiency (0.2%) (7). In another case series, Wang et al. reported that the patients with the aforementioned comorbidities were also more likely to become critically ill and be admitted to the ICU (8), while according to the CCDC data, their case fatality rate was much higher compared to the overall rate of 2.4% (6). The same conclusions were also drawn for older patients (aged 70 and above). These associations were later confirmed in a large study of 1,590 Chinese patients (9). In addition, a more rapid disease progression from symptom onset to death has been described in the elderly (10).

The potential implications of these findings for abdominal organ transplant candidates and recipients are particularly evident. Transplant recipients are most often on life-long immunosuppressants, which predispose them to infections, while transplant candidates usually have a combination of underlying comorbidities and tend to be older compared to the general population. Renal transplant candidates on dialysis are repeatedly undergoing hemodialysis sessions in centers permitting potential exposure and re-exposure of this vulnerable to the virus population with the above-mentioned comorbidities (11). Liver candidates are also at higher risk as decompensated cirrhotics are more prone to infections in general, while most patients with end-stage liver disease awaiting liver transplantation in the U.S. are in their sixth or seventh decade of life (12). That being said, the Wuhan group did not report a higher risk in this population (13). The CDC has, therefore, classified elderly and immunocompromised patients, including transplant recipients, as high-risk patients for severe COVID-19 disease (14). Multiple COVID-19 case series including abdominal organ transplant recipients have recently been published (15–20). The characteristics of these studies are presented in Table 1, while their results are presented in Table 2. A common finding among these studies was that the rates of all adverse outcomes recorded were significantly higher compared to the general population, as expected. The majority of patients were hospitalized and had radiographic evidence of pneumonia. A significant proportion required ICU admission or mechanical ventilation and the case fatality rates recorded were up to 10 times higher compared to those of the general population. However, most of these case series had small samples, thus precluding us from drawing robust conclusions. Another consideration is that confounding factors may have influenced the outcomes in these studies. These include but are not limited to advanced age, high proportion of males, different time intervals between transplantation and infection, and different approaches to immunosuppression tapering or antiviral treatment. All these factors may have adversely affected patient outcomes, and could potentially explain some of the differences between these studies. More robust evidence is needed, in the form of large population-based studies and clinical trials, to further explore these associations and create individualized guidelines for patient management.

**Table 1:** Demographic and management characteristics of COVID-19 case series including abdominal organ transplant recipients.

**References**	**Demographic characteristics**						**COVID-19 Management**			
	**Country**	**Patients**	**Age (mean ± SD)**	**Males**	**Transplanted organs**	**Time from transplantation (median, IQR)**	**Immunosuppression tapered/stopped**	**LPV/r**	**HCQ**	**Tocilizumab**
Fernández-Ruiz et al. (15)	Spain	18	71.0 ± 12.8	14/18 (77.8)	Kidney (8/18), Liver (6/18), Heart (4/18)	9.3 (6.3–16.5)	15/18 (83.3)	9/18 (50.0)	14/18 (77.8)	1/18 (5.6)
Zhu et al. (16)	China	10	45.0 ± 14.0	8/10 (80.0)	Kidney	N/A	9/10 (90.0)	N/A	N/A	N/A
Columbia University (17)	USA	15	50.6 ± 21.4	10/15 (66.7)	Kidney	4.1 (3.2–9.8)	14/15 (93.4)	N/A	13/15 (86.7)	1/15 (6.7)
Pereira et al. (18)	USA	90	57 (46-68)^a^	53 (58.9)	Kidney (46/90), Lung (17/90), Liver (13/90), Heart (9/90), Heart-Kidney (3/90), Liver-Kidney (1/90), Kidney- Pancreas (1/90)	6.64 (2.87–10.61)	42/48 (87.5)^b^, 3/43 (7.0)^c^, 10/56 (17.9)^d^	N/A	62/90 (68.9)	14/90 (15.6)
Akalin et al. (19)	USA	36	57.2 ± 11.2	26/36 (72.2)	Kidney	N/A	24/28 (85.8)^b^, 6/28 (21.4)^c^	N/A	24/28 (85.7)	2/36 (5.5)
Donato et al. (20)	Italy	8	63.0 + 9.4	6/8 (75.0)	Liver	9.7 (3.6–17.1)	N/A	N/A	N/A	N/A

a *Given as median (range)*;

b *Discontinuation or tapering of antimetabolites*;

c *Discontinuation or tapering of corticosteroids*;

d *Discontinuation or tapering of calcineurin inhibitors*.

*SD, standard deviation; IQR, interquartile range; LPV/r, lopinavir/ritonavir; HCQ, hydroxychloroquine; N/A, not available*.

*Numbers in parentheses denote percentages*.

**Table 2:** Clinical symptoms and outcomes of COVID-19 case series including abdominal organ transplant recipients.

**References**	**Symptoms**				**Outcomes**					
	**Fever**	**Cough**	**Myalgia**	**Malaise /Fatigue**	**Hospitalized**	**Pneumonia^a^**	**ICU admission or intubation**	**AKI**	**Death**	**Discharged**
Fernández-Ruiz et al. (15)	15/18 (83.3)	12/18 (66.7)	5/18 (27.8)	4/18 (22.3)	15/18 (83.3)	13/18 (72.2)	2/18 (11.1)	N/A	5/18 (27.8)	8/15 (53.3)
Zhu et al. (16)	9/10 (90.0)	9/10 (90.0)	N/A	9/10 (90.0)	10/10 (100.0)	10/10 (100.0)	0/10 (0.0)	N/A	1/10 (10.0)	8/10 (80.0)
Columbia University (17)	13/15 (86.7)	9/15 (60.0)	2/15 (13.3)	4/15 (26.7)	15/15 (100.0)	9/15 (60.0)	4/15 (26.7)	6/15 (40.0)	1/15 (6.7)	8/15 (53.3)
Pereira et al. (18)	63/90 (70.0)	53/90 (58.9)	22/90 (24.4)	25/90 (27.8)	68/90 (75.6)	68/68 (100.0)	23/90 (25.6) 24/90 (26.7)	N/A	16/90 (17.8)	37/68 (54.4)
Akalin et al. (19)	21/36 (58.3)	19/36 (52.8)	13/36 (36.1)	N/A	28/36 (77.8)	27/36 (75.0)	11/36 (30.6)	6/36 (16.7)	10/36 (27.8)	10/28 (35.8)
Donato et al. (20)	8/8 (100)	N/A	N/A	N/A	5/8 (62.5)	6/8 (75.0)	0/8 (0.0)	N/A	0/8 (0.0)	3/5 (60.0)

a *Confirmed by radiographic findings. ICU, intensive care unit; AKI, acute kidney injury; N/A, not available*.

*Numbers in parentheses denote percentages*.

Respiratory viral infections are common in solid organ transplant recipients and often present atypically (21). Although data for abdominal organ transplant candidates and recipients are still limited, emerging reports have indicated that these patients may present with atypical COVID-19 manifestations. In two individual case reports, two kidney transplant recipients presented with mild gastrointestinal symptoms and no fever. Notably, the patients' temperature remained relatively low (<38.0°C) for several days, and severe symptoms did not manifest until after the first week of illness in both cases (22, 23). In a similar case, poor appetite was the only initial symptom and fever did not develop until 6 days later (24). In two other kidney transplant recipients, fever was present at onset but remained low (<38.0°C) throughout the course of the disease (25, 26). Another kidney transplant recipient did not develop any fever or respiratory symptoms, despite the presence of imaging findings compatible with pneumonia (27). In a case series from the U.S., only 58.3% of the patients (*n* = 21/36) had fever and 52.8% (*n* = 19/36) had cough, the two most common COVID-19 symptoms (19). We can hypothesize that the immunosuppression regimens of these patients might have altered the expected disease course. Besides the presumed increased susceptibility and case fatality, these findings generate additional concerns regarding this patient population. This mild initial course of illness requires very high clinical suspicion and can set the stage for the so-called “super-spreading” events, similar to other viral outbreaks that can put the community at significant risk before appropriate isolation measures are taken (28). The possibility of false negative testing increases this concern (29). An example is a kidney transplant recipient who presented with mild symptoms only and initially tested negative for the virus (25). Transplant recipients are also susceptible to various common respiratory infections due to their immunosuppression regimens. Consequently, when these patients present with respiratory symptoms, the differential diagnosis can become overly complicated and could potentially delay appropriate care (23). This became evident in a case of a liver transplant recipient; COVID-19 diagnosis and appropriate care were delayed due to the patient's atypical presentation and overlapping findings with seasonal influenza (30). In contrast to the previous findings, other case series suggest that COVID-19 in abdominal organ transplant recipients presents the same way as it does in the general population (15–17). The aforementioned concerns for missed cases due to false negative testing or misdiagnosis should be strongly considered when interpreting epidemiologic studies and could be the key to explaining the conflicting nature of the current data.

Additional dilemmas arise regarding the potential discontinuation of immunosuppression regimens to improve their immune response to the infection, which must be weighed against the potential adverse event of transplant rejection. Nevertheless, immunosuppression regimens were fully maintained in many of the reported kidney transplant recipient cases. Notably, the disease remained mild throughout its course and patients recovered uneventfully (25, 26, 31, 32). In all these cases, the authors hypothesized that the immunosuppression regimens may have prevented the overt immune response, manifesting as a “cytokine storm,” that is believed to be responsible for many of the severe manifestations of the disease, such as acute respiratory distress syndrome and multi-organ failure (33). In a report from an Italian transplant center, fully immunosuppressed patients experienced positive outcomes, while three patients on minimal immunosuppression died due to COVID-19 (34). Calcineurin inhibitors, in particular, may also interfere with the life cycle of SARS-CoV-2 (35). However, maintaining immunosuppression may come at the cost of fatal nosocomial infections, as in the case of a liver transplant recipient (36). In addition, confounding factors such as metabolic abnormalities of long-term transplant recipients may be responsible for worse outcomes in patients with low immunosuppression status (34). Cases of mild, uncomplicated disease course have also been described in spite of immunosuppressant discontinuation (22, 30, 37). However, this practice is not risk-free as shown in a liver transplant recipient who entered a temporary state of rejection after immunosuppressant discontinuation as part of his COVID-19 management (38).

The interactions between immunosuppressants and antiviral medication give rise to additional concerns. Tacrolimus is a drug often used after kidney and liver transplantation and is metabolized by CYP3A4. Severe toxicity can occur, as protease inhibitors inhibit this enzyme (39); this includes lopinavir and ritonavir, which are used together as one of the standard regimens for the treatment of COVID-19. A dangerous interaction of this kind was described in a kidney transplant recipient, while in other cases careful dosage adjustments were made, immunosuppressants were discontinued or antivirals were omitted entirely to prevent this adverse reaction (15, 25–27, 32, 36). Similar interactions may occur with many other immunosuppressants metabolized by this pathway. In some cases, immunosuppressants were decreased or discontinued and corticosteroids were initiated or their dosages were increased, in an attempt to prevent adverse drug interactions and disease outcomes, while simultaneously avoiding graft rejection (30, 32, 36). This practice still remains controversial. Cumulative data show that corticosteroid use is associated with worse outcomes in COVID-19, similar to SARS (40, 41). The limited and conflicting data currently prevent us from making any definitive conclusions about the role of corticosteroids and other immunosuppressants during the management of transplanted COVID-19 patients. The Beijing working group for liver transplantation currently recommends that immunosuppressants should not be discontinued unless severe disease develops and drugs that alter their concentrations, including lopinavir/ritonavir, should be avoided due to lack of evidence for their efficacy (42).

## What Does the Past Have To Say?

It is not the first time that humanity faces this kind of threat. Within the last two decades, two similar viral outbreaks have occurred, namely the SARS-CoV outbreak in 2003 and the Middle East Respiratory Syndrome coronavirus (MERS-CoV) in 2012. Both of them, along with SARS-CoV-2, share remarkably similar characteristics, such as their taxonomy (all being coronaviruses), zoonotic origin, direct and indirect human-to-human transmission, pathogenicity, and clinical manifestations (43, 44). Despite their smaller scale, these outbreaks can teach us valuable lessons about the possible effects and the management of the current situation.

The detrimental effects of a global viral outbreak on abdominal organ transplant programs were observed during the SARS-CoV (2003) outbreak. According to a report from a liver transplant program in Hong Kong, transplantations had to be held off due to a combination of fear for community spread, lack of ICU beds, and doctors placed in quarantine. As a result, transplant candidates died while on the waiting list, and recipients missed elective follow-up appointments in fear of being infected (45). Similarly to SARS-CoV-2, the underlying comorbidities and immunocompromised status of transplant recipients may predispose them to high viral burdens of SARS-CoV and atypical clinical presentations (46, 47). The same pattern has also been observed during the MERS-CoV epidemic in renal transplant recipients (48).

Interestingly, massive community spread can occur before appropriate isolation measures are taken, as it has been previously demonstrated in a liver transplant recipient in Toronto (49). Transmission from donors remains another serious concern, which resulted in the development of appropriate screening tools to classify donors according to their infection risk, based on previous history and clinical parameters. Similar protocols were established for potential recipients (49).

## Abdominal Organ Transplantation During the COVID-19 Pandemic

As COVID-19 rapidly evolved into a full-blown pandemic, transplant organizations and services around the globe promptly responded by issuing guidelines and taking appropriate measures to mitigate the risk of transmission between patients and medical personnel. These guidelines address three potential standpoints the epidemic confronts transplantation systems with; first, the risk of donor-derived SARS-CoV-2 infection, which although has not been reported thus far in neither organ or blood product recipients, extensive donor screening protocols have been implemented in many transplant centers in pandemic areas. Second, the risk of nosocomial COVID-19 infection of the living donor and the transplant candidate during the transplant hospitalization as the pandemic increases the fraction of hospitalized patients being infected. This is more relevant for the transplant candidate (as they become recipient) given immunosuppression initiation during the transplant hospitalization. Third, the system-related risks as the allocated resources to transplantation are challenged by the system-wide need for managing the epidemic, including but not limited to hospital staffing, beds (regular and ICU), and blood products, thus affecting the availability of such resources for recipients and deceased donors.

In a Chinese transplant center, an extensive screening protocol has been established for both potential donors and recipients, as well as their families and includes their contact and travel history, clinical and radiological findings, and SARS-CoV-2 laboratory testing. In addition, strict precaution measures are being taken by both patients and medical professionals (50). Similar measures were applied in a transplant program located in a heavily affected area in Italy (51). Organizations including the American Society of Transplantation (AST), the American Society of Transplant Surgeons (ASTS), The Transplantation Society, the Organ Procurement and Transplantation Network, and the Association for Organ Procurement Organizations have all issued similar recommendations (52–56). These recommendations are presented in Table 3. All of them can be summarized as an urgent call for transplant services to adopt strict protocols for the selection and testing of both donors and prospective recipients, along with appropriate isolation measures. Specifically, the AST has developed an algorithm, in order to stratify potential donors according to their SARS-CoV-2 infection risk and currently suggests that only low-risk donors be considered for organ procurement, in addition to intermediate-risk donors under specific circumstances (52). Canada has already implemented this practice, in a similar manner to the SARS (2003) outbreak, as previously mentioned (49, 57). The ASTS recommends SARS-CoV-2 testing on all deceased donors and advises against traveling of the donor organ recovery team, suggesting that organs should be recovered locally instead. If travel is necessary, extreme precautions measures should be taken (52). These proposals strongly reflect the severity of the situation, as ASTS prioritizes measures to decrease transmission in spite of their potential impact on current quality standards for organ recovery. Donor availability may sharply decrease as a result of these restrictions, as documented by a liver transplant center in Italy (58). An important consideration about the current guidelines is that there is no true consensus between transplant organizations globally for any aspect of solid organ transplantation in regards to COVID-19 (59). The wide variety of different and often conflicting approaches to patient management reflects the current lack of data to support a standardized approach with unanimous support by the scientific community. It becomes clear that the transplant community is in great need for more data, not just to understand the effects COVID-19 in transplant recipients, but more importantly to orchestrate a coordinated response based on evidence rather than hypotheses.

**Table 3:** Summary of recommendations from various organizations regarding abdominal organ transplant donors, candidates and recipients.

**Population**	**Subject**	**Current recommendations**	**Endorsed by**
Deceased donors	COVID-19 testing	Routine testing of donors only in areas with significant ongoing community transmission	TTS
		Routine testing of donors with epidemiological or clinical risk factors	AST
		Routine testing of all donors	ASTS
	Testing method	Both upper (nasopharyngeal/oropharyngeal swab) and lower airway samples (BAL)	AST, TTS
		Lower airway sample (BAL)	ASTS
	Exclusion from donation	COVID-19 patients	AST, ASTS
		High-risk patients according to travel or contact history	AST, TTS
		High-risk patients according to clinical symptoms	AST
		Intermediate risk patients according to travel/contact history or clinical symptoms and unavailable COVID-19 testing (only if intestines are used)	AST
	Donation suspension	Tiered suspension should only be considered in countries with widespread transmission	TTS
		May need to be considered for non-urgent cases	AST
		Should be considered on a case-by-case basis	ASTS
Living donors	COVID-19 testing	Routine testing of donors with epidemiological or clinical risk factors	AST
		Routine testing of donors if available	TTS
		Routine testing of all donors	ASTS
	Testing method	Both upper (nasopharyngeal/oropharyngeal swab) and lower airway samples (BAL)	TTS
		Upper (nasopharyngeal/oropharyngeal swab)	AST, ASTS
	Exclusion from donation	Any person with respiratory symptoms or fever^a^	AST, ASTS, TTS
		Any person with high-risk travel or contact history^a^^,^ ^b^	AST, TTS
		COVID-19 patients	AST, ASTS
	Donation suspension	Should be considered for non-urgent cases	AST, ASTS, TTS
Candidates	Consultations	Telemedicine or phone consultations should be utilized whenever possible	AST, ASTS
	COVID-19 testing	If the patient is considered high-risk for COVID-19 exposure and testing is available	AST
	Transplantation deferment	For COVID-19 patients until ≥ 2 negative samples and symptom resolution	AST
		Temporary suspension of all non-urgent cases may be considered	AST, ASTS, TTS
Recipients	Travel	Avoid all travel in areas with SARS-CoV-2 transmission	TTS
		Avoid cruise ships	TTS
		Avoid all non-essential travel	AST
	Medication	Patients should carry an extended supply of their medicines	AST, ASTS
	Symptom development	Patients should call their transplant centers and avoid going to clinics	AST, ASTS, TTS

*BAL, Bronchoalveolar lavage; TTS, The Transplantation Society; AST, American Society of Transplantation, ASTS, American Society of Transplant Surgeons*.

a *The AST recommends deferment for 28 days beyond symptom resolution plus ≥ 2 negative SARS-CoV-2 tests if high-risk*.

b *TTS recommends deferment for 14 days*.

Meanwhile, the lack of data surrounding many aspects of COVID-19 disease and its effects on transplant patients further complicate the situation and may necessitate the application of more drastic measures. The viability of SARS-CoV-2 in blood or various organs remains unclear, and this could significantly affect the donor-to-recipient transmission risk. In a preliminary report, viral RNAemia was found in 15% of the 41 tested patients (5). The angiotensin-converting enzyme 2 human cell receptor, which is implicated in the pathogenicity of SARS-CoV-2, has been found to be highly expressed in proximal tubule cells of the kidney, but minimally in liver cells (60). On the other hand, liver inflammation attributed to COVID-19 has been described in a liver transplant recipient, suggesting that the virus can affect the liver and generating concerns about potential donor-recipient transmission (61). Additional data regarding these aspects of SARS-CoV-2 could further individualize guidelines for liver and kidney transplantation.

Current recommendations must be routinely revised, as the COVID-19 outbreak is rapidly escalating. Kumar et al. recently proposed a four-staged approach to restrict the activity of solid organ transplantation programs according to the severity of the outbreak and its burden on healthcare (57). However, more radical approaches are also being considered. The AST has already warned that all non-urgent transplantations might be temporarily suspended at any time in an effort to control the situation. England has already moved in this direction by suspending all elective surgeries over the next 3 months (62). More recently, India officially suspended all non-urgent liver transplantations (63). It is likely that many countries will soon follow this strategy, if they have not done so already. This situation will lead to ethical dilemmas, where the benefits of saving a patient's life must be weighed against the risk of disease transmission to the patient and the community. The lack of reliable data regarding immunosuppressed patients, including transplant recipients, has sparked further controversy about this decision. The current notion that these patients are at increased risk for severe disease or death and the effectiveness of shutting down transplant programs have recently been disputed (64). Nevertheless, additional factors beyond the immunocompromised status of transplant recipients, including but not limited to their age, underlying comorbidities, and type of transplant, must be taken into account during the decision-making process. Another concern is that inequalities to healthcare access, including those surrounding the abdominal organ transplantation process, may be amplified by the restrictions put in place due to the pandemic (65).

## Discussion

In contrast to other infectious diseases where only the transplant recipient is at risk, SARS-CoV-2 could rapidly spread amongst medical personnel, resulting in serious consequences to the community (66). As a result, it becomes imperative that both patients and medical professionals strictly adhere to all appropriate safety measures geared toward minimizing transmission, in order to ensure that transplant programs can continue to operate uninterrupted for as long as possible, without placing the patients or the community at risk. However, transplant organizations must remain vigilant and frequently update their recommendations. At the same time, administrative authorities at a local, regional, and nationwide level must be ready to respond appropriately and take all measures necessary to ensure the safety of public health, including temporary discontinuation of all non-urgent transplantations.

## Author Contributions

IZ and DG conceived the original research idea. SE and MH performed the literature search and drafted the original manuscript. IZ, DG, NE, and GT critically reviewed and edited the manuscript. All authors have read and approved the submitted manuscript version.

## Conflict of Interest

The authors declare that the research was conducted in the absence of any commercial or financial relationships that could be construed as a potential conflict of interest.
